# Th17 cells and their cytokines serve as potential therapeutic target in experimental autoimmune neuritis

**DOI:** 10.1002/brb3.1478

**Published:** 2019-11-19

**Authors:** Shuping Liu, Yin Liu, Zheman Xiao, Sijia Pan, Qiaoyu Gong, Zuneng Lu

**Affiliations:** ^1^ Department of Neurology Wuhan University, Renmin Hospital Wuhan China

**Keywords:** experimental autoimmune neuritis, IL‐17, receptor‐related orphan receptor‐gamma‐t

## Abstract

**Background:**

Accumulating evidence has pointed that T helper 17 cells and their cytokines are pathogenic in Guillain–Barré syndrome (GBS). However, little is known concerning the IL‐17 expression change trend during the whole course of disease, and whether drugs specially targeting Th17 cells or their cytokines have potential effects on experimental autoimmune neuritis (EAN) is uncertain.

**Methods:**

We explored the IL‐17 and receptor‐related orphan receptor‐gamma‐t (RORγt) expression change trends in EAN rats to identify the stage of effect of Th17 pathway in EAN, and further, we investigated the effect of RORγt inhibitors by assessing clinical score, histological staining, and IL‐17 and RORγt expression change trends in serum and tissues.

**Results:**

The expression level of IL‐17 and RORγt in serum and tissues increased with the progression of the disease in the EAN group and decreased after the disease reaching its peak. RORγt‐IN‐1 treatment strikingly reduced the neurological deficits by ameliorating inflammatory cell infiltration, deceased the serum IL‐17 and RORγt levels, and further downregulated the expression of IL‐17 and RORγt mRNA in spleen, lymphnodes, and sciatic nerve.

**Conclusions:**

Th17 cells and their cytokines are closely associated with the onset of GBS and the novel RORγt inhibitors may be prospective strategies in treating GBS.

## Graphical abstract

Th17 cells and their cytokines are closely associated with the onset of Guillain–Barré syndrome (GBS) and the novel receptor‐related orphan receptor‐gamma‐t inhibitors may be prospective strategies in treating GBS.

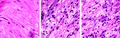

## 1 INTRODUCTION

Guillain–Barré syndrome (GBS) is a heterogeneous, immune‐mediated illness that afflicts the peripheral nervous system (Doorn, Ruts, & Jacobs, 2008). Pathologically, GBS is mainly divided into demyelinating and axonal form, with demyelination form predominating in most countries and regions (Wakerley & Yuki, 2015). Although plasma exchange and intravenous immunoglobulin were proved to be effective, 20% of GBS patients still suffered from severe disability, and approximately 2%–10% of them died prematurely (Hughes & Cornblath, 2005). Therefore, more effective therapeutic strategies dependent on in‐depth understanding the pathogenesis of GBS are urgently needed.

Experimental autoimmune neuritis (EAN) is a classical model that mirrors demyelinating form of human GBS in clinical, immunological, histopathological, and electrophysiological aspects, and has been widely applied to investigate immunological therapy and disease mechanisms of GBS (Gold, Hartung, & Toyka, 2000). Pathologically, EAN is characterized by breakdown of the blood–nerve barrier, accumulation of auto‐reactive T cells and macrophages, and segmental demyelination in the peripheral nerves (Zhang, Zheng, & Zhu, 2013). In terms of pathogenesis, EAN had been previously attributed to Th1 cells and their cytokines, such as interferon‐γ (IFN‐γ) or tumor necrosis factor α (TNF‐α; Kieseier et al., 2004; Zhu, Bai, Mix, & Link, 1997). Recently, accumulating evidences from clinical studies suggested a pathogenic role of Th17 cells and their cytokines in EAN, and this pathological role might largely be fulfilled through the secretion of IL‐17, which is a hallmark cytokine of Th17 cells (Wang et al., 2014; Zhang et al., 2012). However, there have been limited numbers of IL‐17‐related EAN studies and little is known concerning the IL‐17 expression change trend during the whole course of autoimmune injury of the PNS. Thus far, we investigated the changes in expression of IL‐17 and retinoic acid receptor‐related orphan receptor‐gamma‐t (RORγt), the transcription factor of Th17 cells (Ivanov et al., 2006), in serum, immune tissues and disease target organs.

Considering that using drugs specially targeting Th17 cells or their cytokines to show the elimination or alleviation of GBS/EAN can not only further verify the pathogenic role of Th17 cells and their cytokines in GBS/EAN, but also pave the way for finding new strategies of GBS, we further assessed the effect of a novel ROR gamma‐t‐inhibitor‐1 (RORγt‐IN‐1) with good oral bioavailability and central nervous system (CNS) penetration in treating EAN (Wang et al., 2015).

## 2 MATERIALS AND METHODS

### 2.1 Animals

Female Lewis rats, 6–8 weeks old (body weight 150–180 g), purchased from Beijing Vital River Laboratory Animal Technology Co., Ltd. were used in the study. The rats were kept under pathogen‐free conditions at the local animal house and fed with standard rat chow and water ad libitum. All the experimental protocols were approved by the Institutional Animal Care and Use Committee of Wuhan University and conducted in accordance with the Declaration of the National Institutes of Health Guide for Care and Use of Laboratory Animals and the People's Republic of China animal welfare legislations. The minimum number of animals was used to result in meaningful interpretation of data, and animal pain and discomfort were kept to a minimum level.

### 2.2 Induction of EAN

Experimental autoimmune neuritis was induced by subcutaneous injection into the tail heel of the rats with 200 μl inoculum containing 400 μg P2^57‐81^ (F‐K‐N‐T‐E‐I‐F‐K‐L‐G‐Q‐E‐F‐E‐E‐T‐T‐A ‐D‐N‐R‐K‐T‐K; GL Biochem Ltd.), 100 μl incomplete Freund's adjuvant (FIA; Sigma), 100 μl PBS, and 2 mg mycobacterium tuberculosis (strain H37RA; Difco).

### 2.3 Assessment of neurologic symptoms

Changes in body weight were recorded daily, and clinical symptoms of disease were monitored by two independent investigators. Clinical scores were assessed immediately before immunization (day 0) and thereafter everyday until the rats were sacrificed. The severity of clinical symptoms was scored as follows: 0, normal; 1, reduced tonus of tail; 2, limp tail; impaired righting; 3, absent righting; 4, gait ataxia; 5, mild paresis of the hind limbs; 6, moderate paraparesis; 7, severe paraparesis or paraplegia of the hind limbs; 8, tetraparesis; 9, moribund; and 10, death.

### 2.4 Blood samples and tissues

Peripheral blood samples (1–1.5 ml) were collected on day 0, day 7, day 14, day 17, and day 28 postimmunization (p.i.) if necessary from caudal vein directly into containers for enzyme‐linked immunosorbent assay (ELISA), as were control samples.

Rats were sacrificed, and then spleens and inguinal lymphnode were quickly removed under aseptic conditions and stored in liquid nitrogen until RNA isolation. Bilateral sciatic nerves were excised close to the lumbar spinal cord, one side sciatic nerves were fixed in 10% paraformaldehyde and embedded in paraffin for histopathological assessment, and the other side were used for quantitative real‐time polymerase chain reaction (RT‐PCR) analysis.

### 2.5 Histopathological assessment

Multiple longitudinal sections (5 μm) of sciatic nerves were stained with hematoxylin and eosin for evaluation of the inflammatory cells by light microscopy.

### 2.6 Enzyme‐linked Immunosorbent assay

Enzyme‐linked immunosorbent assay was applied to measure IL‐17 and RORγt in serum. Blood samples after static placement (40 min) were subjected to centrifugation (4°C) at 1006 *g* for 20 min, and supernatants were then collected. IL‐17 was determined by the rat IL‐17 ELISA Kit (Elabscience Biotechnology Co., Ltd), and RORγt by rat RORγt ELISA Kit (Enzyme‐linked Biotechnology). All procedures were done according to the manufacturer's instructions, and each sample was assayed in duplicate. The spectrophotometry of panels was read at 450 nm and calculated according to the standard curve.

### 2.7 Quantitative real‐time polymerase chain reaction

Total RNA from lymphnodes, spleen, and sciatic nerves was extracted using TRIzol (Ambion), and reverse transcription was carried out with a Reverse Transcriptase Kit (Vazyme Biotech). All procedures were performed in strict accordance with each manufacturer's instructions. Real‐time PCR was performed with the following primers: RORγt (forward 5′‐ACCAACCTCTTC TCACGGG‐3′, reverse 5′‐CTTCCATTGCTCCTGCTTTC‐3′), IL‐17 (forward 5′‐CTTCTGTGATCT GGGAGGCA‐3′, reverse 5′‐GGCGGACAATAGAGGAAACG‐3′), and β‐actin (forward 5′‐CACGATGGAGGGGCCGGACTCATC‐3′, reverse 5′‐TAAAGAC CTCTATGCCAACACAGT‐3′). PCR was run in a ViiA 7 real‐time PCR System (Applied Biosystems) using a general SYBR green fluorescence detection for 10 min at 95°C, followed by 40 cycles each of 15 s at 95°C, at 60°C for 1 min. The calculation of relative quantitative expression was done using 2-ΔΔCT method.

### 2.8 Part 1

Rats in the same batch were randomly assigned to the EAN group 1 (rats were sacrificed on day 7 p.i.), EAN group 2 (rats were sacrificed on day 17 p.i.), EAN group 3 (rats were sacrificed on day 28 p.i.), and the control group (rats were sacrificed on day 28 p.i.). Among which, the control group rats were injected with an equal volume of emulsion without the P2 peptide. The main purpose of this part was to investigate IL‐17 expression change trend during the whole course of EAN.

### 2.9 Part 2

Rats in the same batch were modeled and were randomly assigned to the RORγt‐IN‐1‐treated group and the control group. Of which, the control group EAN rats received an equal volume of PBS. The novel RORγt‐IN‐1 administration has been investigated in experimental autoimmune encephalomyelitis, and it can be concluded that significant therapeutic effect can be obtained at doses of 3 mg kg^−1^ day^−1^ (Wang et al., 2015). To determine whether this kind of RORγt inhibitor protected against the development of EAN, RORγt‐IN‐1 (at doses of 3 mg kg^−1^ day^−1^) was administered to experimental group rats via intragastric injection from day 0 to 28 p.i. The dose was prepared on the day of administration in suspension (dimethyl sulfoxide, DMSO/1% methylcellulose = 1:99 as the vehicle) at a concentration of 0.6 mg/ml. We observed these rats for 28 days, mainly for the purpose of preliminarily assessing the impact of RORγt‐IN‐1 on disease progression, and to explore the serum IL‐17 and RORγt change curve under the action of drugs.

### 2.10 Part 3

Rats in the same batch were modeled and were divided randomly into the RORγt‐IN‐1‐treated group, positive control group, and the negative control group (*n* = 6). Positive control EAN rats received the same volume injections of vehicle (dimethyl sulfoxide, DMSO), and the negative control EAN rats received the same volume of PBS. The rats were sacrificed at the fastigium of clinical symptoms of EAN (day 17 p.i.), and the main purpose of this part was to explore the effect of RORγt‐IN‐1 on the expression of IL‐17 and RORγt mRNA in tissues at the peak time of diseases. The reason for setting positive control in this part was to exclude the toxic effect of low‐dose DMSO on rats.

### 2.11 Statistical analysis

Statistical analyses were performed using SPSS version 22.0 (IBM Corp). Continuous variables were presented as the mean ± standard deviation and compared using *t* tests and analysis of variance (ANOVA). Differences among the groups were analyzed using repeated‐measures ANOVA. Differences were considered statistically significant at a *p*‐value <.05.

## 3 RESULTS

### 3.1 Part 1

#### 3.1.1 Clinical symptoms and body weight

**Figure 1:**
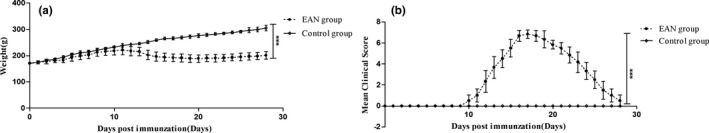
Weight (a) and clinical score (b) change curve of both groups (EAN group and control group, *n* = 6, respectively). ***p < .001

All the immunized rats exhibited the clinical syndromes of EAN, while the rats in the control group grew well throughout the study (Figure 1b). Varying degrees of clinical signs of disease were observed starting from day 10 p.i. in EAN group, mainly with progressively decreasing body weight, listlessness and decreased activity, and reaching the peak on the 16th to the 17th day. Accordingly, rats in the two groups had significantly different weight changing curve, with rats in the control group gaining weight gradually, while EAN group rats losing weight after 11 days p.i. (*p* < .001; Figure 1a). At the peak of the disease, the clinical scores of rats in the EAN group were significantly higher than that in the control group (*p* < .001; Figure 1b).

#### 3.1.2 Serum IL‐17 and RORγt change trends

**Figure 2:**
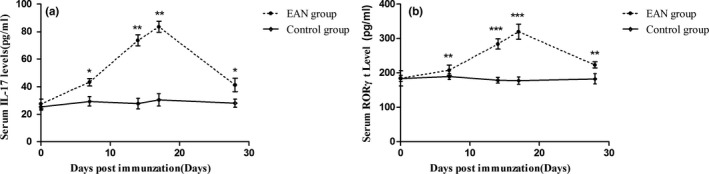
Serum levels of IL‐17 (a) and RORγt (b) increased with the progression of the disease in the EAN group (*n* = 6), while no significant fluctuation was identified in control group (*n* = 6). *p < .05, **p < .01, and ***p < .001

Serum IL‐17 and RORγt level increased with the progression of the disease in the EAN group and decreased after the disease reaching its peak, while no significant fluctuation was identified in control group. The serum IL‐17 in the EAN group was significantly higher than that in the control group (*p* < .05 on day 7 p.i., *p* < .01 on day 14 p.i., *p* < .001 on day 17 p.i., and *p* < .05 on day 28 p.i.), as was the serum RORγt level (*p* < .01 on day 7 p.i., *p* < .001 on day 14 p.i., *p* < .001 on day 17 p.i., and *p* < .01 on day 28 p.i.; Figure 2).

#### 3.1.3 IL‐17 and RORyt mRNA expression change trends

**Figure 3:**
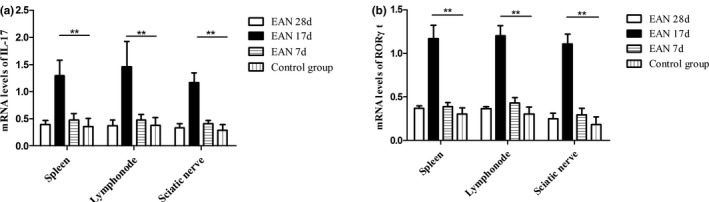
IL‐17 (a) and RORγt (b) mRNA expression level in spleen, lymph node, and sciatic nerve of each group (EAN group and control group, *n* = 6, respectively). **p < .01

Upregulation of IL‐17 and RORγt in EAN rats was further tested at the mRNA level. The expression of IL‐17 and RORγt mRNA in tissues of EAN rats increased with the progression of the disease and decreased after the disease reaching its peak. Compared with the rats in control group, the expression of IL‐17 and RORγt mRNA at the peak time in spleen (*p* < .01 and *p* < .001, respectively), lymph node (*p* < .01, *p* < .001 respectively), and sciatic nerve (*p* < .01 and *p* < .001, respectively) of the EAN group rats dramatically increased (Figure 3).

### 3.2 Part 2

#### 3.2.1 RORγt‐IN‐1 ameliorates neurological symptoms of EAN

**Figure 4:**
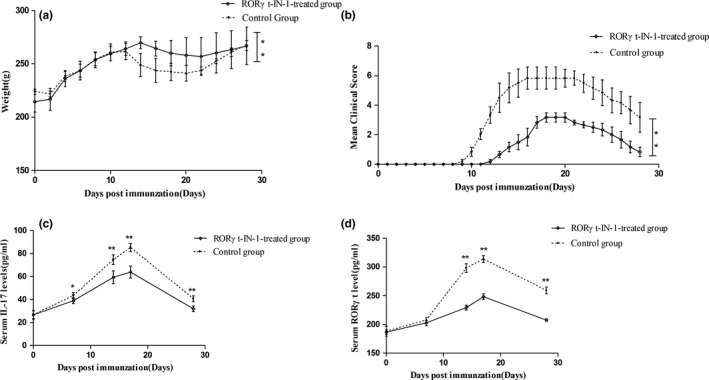
Weight (a), clinical score (b), serum IL‐17 (c), and serum RORγt (d) change curve of RORγt‐IN‐1‐treated EAN rats (*n* = 6) and the control EAN rats (*n* = 6). Lewis rats were immunized with P2^57‐81^ in incomplete Freund's adjuvant and monitored for initiation and development of EAN. RORγt‐IN‐1‐treated EAN rats were treated with RORγt‐IN‐1 at daily doses of 3 mg/kg from day 0 to 28 p.i., and control EAN rats received the same volume of PBS. *p < .05, **p < .01

RORγt‐IN‐1, a novel RORγt inhibitors, not only delayed the onset of EAN, but also significantly decreased the neurological severity of EAN (Figure 4a,b). The mean day of EAN onset was day 13.5 ± 1.38 in the RORγt‐IN‐1‐treated group and day 11.1 ± 0.75 in the control EAN group. The rats in RORγt‐IN‐1‐treated group exhibited significantly lower clinical scores from day 10 p.i. compared with the rats in control EAN group (*p* < .01; Figure 4b).

#### 3.2.2 RORγt‐IN‐1 reduces the levels of RORγt and IL‐17 in serum

The serum levels of IL‐17 in EAN rats gradually increased with disease progression and decreased after the disease reaching its peak (Figure 4c), as was the serum RORγt levels (Figure 4d). In addition, both IL‐17 and RORγt in serum at 14, 17, and 28 day p.i. were significantly lower in RORγt‐IN‐1‐treated rats when compared with control EAN rats (IL‐17: *p* < .01, *p* < .01, and *p* < .01, respectively; RORγt: *p* < .01, *p* < .01, and *p* < .01, respectively).

### 3.3 Part 3

#### 3.3.1 RORγt‐IN‐1 protects against EAN‐induced peripheral nerve injury

**Figure 5:**
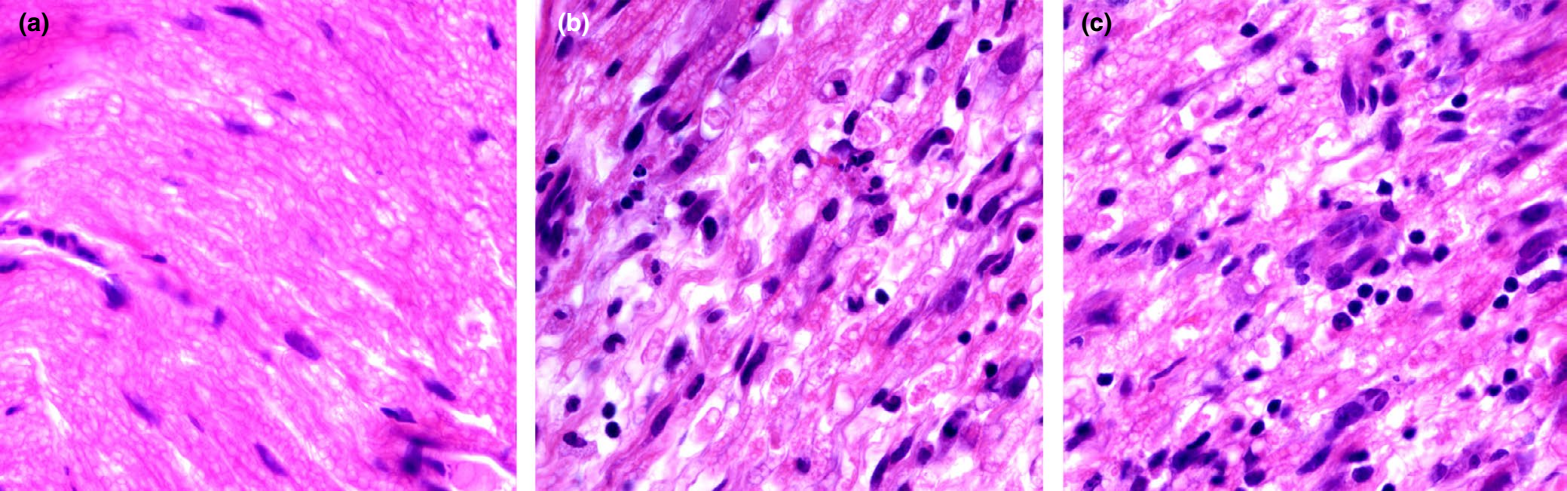
Sciatic nerve sections from RORγt‐IN‐1‐treated group (a, *n*=6) showed fewer inflammatory cells infiltration; Sciatic nerve sections from DMSO‐treated group (b, *n*=6) and control group (c, *n*=6) showed large amount of inflammatory cells(HE×400). Lewis rats were immunized with P2^57‐81^ in incomplete Freund's adjuvant. RORγt‐IN‐1‐treated EAN rats were treated with RORγt‐IN‐1 at daily doses of 3 mg/kg from day 0 to 17 p.i., DMSO‐treated EAN rats received the same volume of DMSO, and control EAN rats received the same volume of PBS

To confirm whether the benefits of RORγt‐IN‐1 against clinical symptoms of EAN were due to the decreased inflammation in the PNS, the sciatic nerves of EAN rats were examined histologically just after the peak of the clinical course of EAN. RORγt‐IN‐1‐treated group displayed fewer localized inflammatory cells and less severe demyelination than the DMSO control group and the negative control group. These data indicated that the mild EAN clinical scores in the groups of RORγt‐IN‐1 administration were associated with the decreased numbers of inflammatory cells in the PNS (Figure 5).

#### 3.3.2 RORγt‐IN‐1 attenuates the expression of IL‐17 and RORγt mRNA

**Figure 6:**
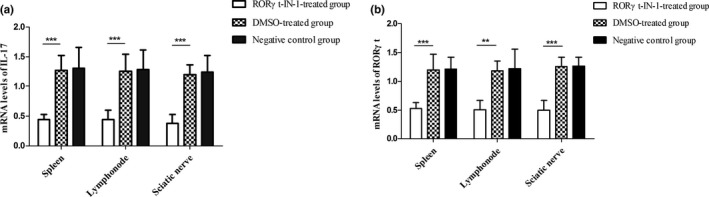
IL‐17 (a), RORγt (b) mRNA expression in spleen, lymphonde and sciatic nerve of each group (RORγt‐IN‐1‐treated group, DMSO‐treated group and control group, *n*=6 respectively). Lewis rats were immunized with P2^57‐81^ in incomplete Freund's adjuvant. RORγt‐IN‐1‐treated EAN rats were treated with RORγt‐IN‐1 at daily doses of 3 mg/kg from day 0 to 17 p.i., DMSO‐treated EAN rats received the same volume of DMSO, and control EAN rats received the same volume of PBS. **p < .01 and ***p < .001

Spleen, lymphnode, and sciatic nerves from RORγt‐IN‐1‐treated rats showed a significantly reduced expression of IL‐17 mRNA, compared to those tissues from DMSO‐treated group (*p* < .001, *p* < .001 and *p* < .001 respectively). The similar pattern was observed for RORγt mRNA, with RORγt‐IN‐1 treatment strikingly decreasing the expression of RORγt mRNA in spleen, lymphnode, and sciatic nerve (*p* < .001, *p* < .001, and *p* < .001, respectively; Figure 6).

## 4 DISCUSSION

Th17 cells, which is considered as a subset of CD4+ T cells, have recently been found to play a critical role in the development of autoimmune disease (Bettelli, Korn, Oukka, & Kuchroo, 2008; Kurts, 2008). IL‐17, also named IL‐17A, is mainly expressed by Th17 cells and involved in inducing proinflammatory response through directly stimulating epithelial cells, endothelial cells, and fibroblasts to produce proinflammatory cytokines and chemokines (Liao, Huang, & Goetzl, 2007). As a transcription factor specific to Th17 inflammatory pathways, RORγt plays a vital role in Th17 cell differentiation (Ivanov et al., 2006). The high IL‐17 levels in plasma of GBS patients demonstrated that Th17 cells might play a pivotal role in the pathogenesis of GBS (Han et al., 2014; Li, Yu, Li, Zhang, & Jiang, 2012).However, limited information is available about the relationship between IL‐17 change trend and disease evolution of EAN. In order to further clarify the roles of Th17 inflammatory pathways in the pathogenesis of EAN, we firstly analyze the accumulation of IL‐17 and RORγt concentration in serum of EAN rats at different time points, and their correlation with pathological progress of EAN. Our study revealed that the serum levels of IL‐17, as well as RORγt, increased progressively from the first week postimmunization and reached higher value at the peak of EAN. Given that varying degrees of clinical signs of disease were observed starting from day 10 postimmunization in EAN group, these data suggested that the immune response to the myelin in peripheral nerves has been activated in vivo before the clinical symptoms exhibited. That's why we initiated the RORγt treatment just after immunization. In addition, the fact that IL‐17 and RORγt expression in spleen, lymph node, and sciatic nerve of EAN rats was strikingly higher than that in control group at the peak of symptoms further suggested that IL‐17 may have pro‐inflammatory effect and may be closely related to EAN. Of note was that, control group rats exhibited no symptoms and pathological examination showed no obvious changes as well. Nonetheless, we still found a small amount of expression of IL‐17 and RORγt mRNA in tested tissues. The possible reason was that the mycobacterium tuberculosis composition in the immunization inoculum stimulated the glial cells and macrophages to produce a few Th17 cells/IL‐17, but the inflammatory response did not expand up due to the lack of specific antigen stimulation (Rojas, Olivier, & Garcia, 2002; Song et al., 2003).

RORγt‐IN‐1 is an agonist of RORγt that has displayed promising protective effects in animal models of multiple sclerosis (Wang et al., 2015). In our preliminary experiment, we have shown for the first time that RORγt‐IN‐1 greatly improved EAN symptoms by reducing the severity of symptoms, delaying the onset of the first signs of EAN, and decreasing peak neurologic scores. In addition, RORγt‐IN‐1 treatment effectively reduced the serum level of both IL‐17 and RORγt at 14, 21, and 28 day p.i., which was demonstrated by the significantly lower serum levels of IL‐17 and RORγt in RORγt‐IN‐1‐treated rats. Besides, the change trend of IL‐17 in both groups was highly consistent with that of clinical scores, which further validates a pathological contribution of IL‐17 to the development of EAN.

In order to exclude influence of DMSO on the experimental results, we performed more detailed grouping methods. Rats in the same batch were divided randomly assigned to the RORγt‐IN‐1‐treated group, DMSO‐treated group, and the negative control group. Histopathologically, RORγt‐IN‐1 treatment significantly suppressed the accumulation of inflammatory cell infiltration and demyelination when compared to the DMSO group. Our results further revealed that RORγt‐IN‐1 greatly reduced the expression of both IL‐17 and RORγt mRNA at peak time in spleen, lymphnode, and sciatic nerves. Although these data obtained from spleen and lymphnode samples may not exactly represent changes in peripheral nerves in situ, they may nonetheless provide mechanistic insight into the physiological activities underpinning this potentially valuable therapeutic and shed light on the positive clinical outcome we observed.

The beneficial effects of RORγt‐IN‐1 in EAN rats can not only further confirmed the role of Th17 inflammatory pathway in the pathogenesis of EAN, but also provided a new idea for the treatment of EAN/GBS. Although many approaches targeting Th17 cells or their cytokines are in development to treat Th17 cell‐mediated inflammatory or autoimmune diseases, such as multiple sclerosis (Berg & McInnes, 2013; Shiomi & Usui, 2015; Zhu & Qian, 2012). We still consider that targeting Th17 cell development might be superior than blocking a single cytokine, since Th17 cells might exert their pathogenic role by producing different sets of pro‐inflammatory cytokines (Wu, Wang, Liu, Zhu, & Zhang, 2016). Due to the essential role of RORγt in the development of Th17 cells (Skepner et al., 2015), the RORγt inhibitors are expected to be the most valuable prospective strategies.

## 5 CONCLUSIONS

Taken together, Th17 cells and their cytokines are closely associated with the onset of GBS and the novel RORγt inhibitors may be prospective strategies in treating GBS. However, a variety of T helper (Th) subsets (Th1/Th2/Th17/Tregs) and a complex network of cytokines are implicated in the pathogenesis of GBS/EAN. Further studies are needed to provide detailed insights into the roles of these Th and Treg cytokines in the pathogenesis of EAN and their interrelationship.

## CONFLICT OF INTEREST

The authors declare no financial or other conflicts of interest.

## AUTHORS' CONTRIBUTIONS

S.L. and Y.L. performed the experimental research, read a lot of literature, and wrote the first draft; Z.L. had the idea of performing this research, performed the literature search, and critically appraised the draft; S.P. and Q.G. fully assisted the experimental procedure; and Z.X. critically modified the grammar and participated in the statistical process. All authors read and approved the final manuscript.

## ETHICAL APPROVAL

All the experimental protocols were approved by the Institutional Animal Care and Use Committee of Wuhan University and conducted in accordance with the Declaration of the National Institutes of Health Guide for Care and Use of Laboratory Animals and the People's Republic of China animal welfare legislations.

## Data Availability

The datasets generated and/or analyzed during the current study are available from the corresponding author on reasonable request.
